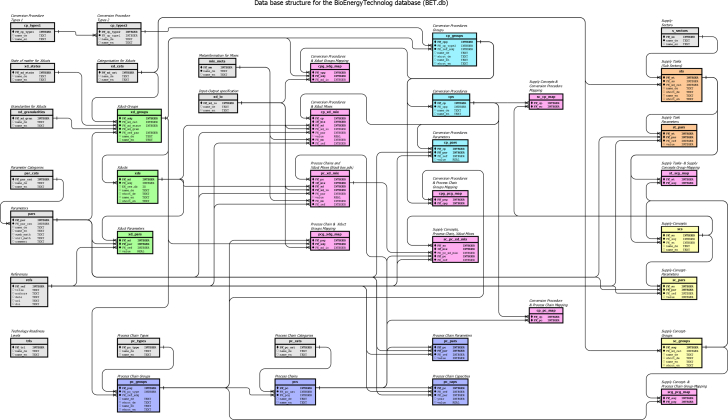# Advanced SQL-Database for bioenergy technologies - A catalogue for bio-resources, conversion technologies, energy carriers, and supply applications

**DOI:** 10.1016/j.heliyon.2024.e25434

**Published:** 2024-02-01

**Authors:** Martin Dotzauer, Kai Sven Radtke, Matthias Jordan, Daniela Thrän

**Affiliations:** a DeutschesBiomasseforschungszentrum gemeinnützige GmbH, Torgauer Straße 116, 04347, Leipzig, Germany; b Helmholtz Centre for Environmental Research – UFZ, Permoserstraße 15, 04318, Leipzig, Germany

**Keywords:** Technology, Database, Biomass, Catalogue, Bioenergy

## Abstract

Bioenergy is a crucial element of the future energy system with wide range of applications in electricity, heat and transport. A major challenge for the analysis and optimisation of the bioenergy system is the degree of diversity and complexity compared to wind or solar energy. A coherent database for studying the role of bioenergy in the energy system needs to cover the different entities such as bio-resources, conversion procedures and process chains. Since there is no comprehensive data collection for bioenergy so far, we develop a SQLite database by merging several existing datasets and additional information. The resulting Bio-Energy Technology Database (BET.db) provides a consistent set of 141 feedstocks as well as energy carriers, 259 conversion technologies, and 134 energy supply concepts. The proof of concept within a bioenergy system modelling a wide range of technologies for the electricity, heat and transport sectors using the BENOPT model has been successful. By providing a one-stop-shop solution for techno-economic information about on the bioenergy nexus, this blind spot can be avoided for further investigations. The current stage of development is an intermediate prototype that will be developed into a more versatile and interactive web application later on.

## Highlights

• BET.db provide techno-economical key values for resources, processes and products.
• Presented SQL database is a comprehensive information collection of high integrity.
• Contain 141 raw materials, 259 conversion technologies, and 134 supply concepts.
• In depth explanation of the database structure to enable further integration.

## Graphical abstract

**Figure:**
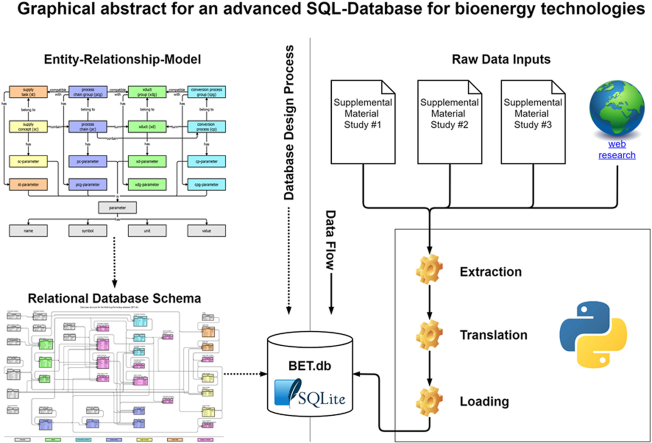

## Abbreviations

**Abbreviation Description**
BENOPT: Bioenergy Optimisation Model
BET.db: Bioenergy Technology Database
CHP: Combined Heat and Power
CP: Conversion Procedure
CPG*: Conversion Procedure Group
ERM: Entity Relationship Model
FAIR: Findable, Accessible, Interoperable, Reusable (research data management paradigms)
GHG: Greenhouse Gas Emission
NET: Negative Emission BackspaceTechnologies
PAR: Parameter
PC: Process Chain
PCG*: Process Chain BackspaceGroup
SC: Supply Concept
SOBIO: System-Optimal Biomass Allocation (a research project)
ST: Supply Task
XD: Xduct (mass or energy flow, which could be an educt as well as a product)
XDG*: Xduct Group

* Upper case letters are used for abbreviations in this manuscript. Italic fonts are used for naming tables and views of the database. Attributes are written with *tablename.attribute* notation.

### 1 Introduction

The global transition to renewable energy is leading to a growing research focus on renewable technologies in general [1,2]. In addition to the main workhorses for electricity generation, namely wind energy and photovoltaics, bioenergy technologies are also becoming increasingly important [3].

The use of biomass for energy and material applications instead of fossil resources can be summarised under the term bioeconomy [4]. In 2012, the European Commission adopted a strategy entitled *"Innovation for sustainable growth: a bioeconomy for Europe”* [5]. In the bioeconomy nexus, potential resources, conversion technologies and the potential products cover a very wide range of individual items [6,7]. However, bioenergy as a sector of the bioeconomy is often produced in complex process chains (PC) compared to other renewable energies such as wind and solar [3,8,9]. Bioenergy process chains start with biomass resources, are delivered via transported intermediates, processed into different energy carriers and finally used in different applications in all energy sectors. The possibility of including potential by-products makes these process chains even more complex.

Considering bioenergy applications in systemic studies such as energy system modelling, investigation of technical and economic parameters for a large number of resources, intermediates, conversion processes, energy carriers and specific products is very challenging. During the data preparation for the System-Optimal Biomass Allocation (SOBIO) project we decided to carry out a preparatory study to collect information for all entities planned to be included in the SOBIO project. Our aim is to organise all the results of the preparatory study within a single and consistent data collection. The main objectives are, firstly, to create a universal data model that is compatible with different types of entities. A second objective was to build a repository that can be accessed by the modelling approach used, in order to streamline the workflow within the SOBIO project and reduce inadvertent errors in maintaining and handling large data sets manually.

The data collection is largely based on technology information related to Germany, for which bioenergy technologies have already been described in previous studies. We integrate several existing data sources with the aim of harmonising data and improving accessibility through a consistent data structure and clearly defined data types. Essential input data are obtained from three main sources, which are described in the following section 4. The detailed process of primary data harmonisation and database structuring is described in section 5.

The data in studies on energy systems with a focus on bioenergy are structured differently. Lauer et al. [10] describe the crucial role of bioenergy in a climate-neutral electricity system in Germany. They provide supplementary information on the technologies studied and the assumptions made about the available quantities of biogenic feedstocks and their properties. This dataset forms the starting point for the electricity and Combined Heat and Power (CHP) technologies in Bioenergy Technology Database (BET.db) and is complemented by many other primary data sources.

Jordan et al. [11] provide an appendix in the supplementary material [12] for a study of the heat sector, which contains techno-economic data sets for CHP and heat technologies, as well as for supply tasks and the combination of both building supply concepts. The dataset is provided as an MS Excel workbook, where each worksheet represents a specific supply task. The header of each worksheet provides a brief description of the selected key values for the supply-task. These include energy consumption per building rather the specific heat sink, annual full load hours and the thermal heat demand. This is followed by a section on the qualitative description of suitable supply concepts.

The actual data is presented in a data table where the columns represent the different supply concepts and the rows are the quantitative data.

Meisel et al. [13] provide supplementary material in the form of an MS Excel workbook in a study on the transport sector with a selection of biofuels process chains. The first worksheet of the MS Excel file documents the input data for the Bioenergy Optimisation Model (BENOPT) of Millinger et al. [[14], [15]] used. The header of the data table consists of two rows for the main feedstock and the technology acronyms. The following rows contain both quantitative techno-economic data and qualitative information on the meta-properties of the technologies considered.

The database was developed within the context of a preparatory study for a specific use case in the SOBIO project. BET.db was specifically designed to be used as a directly accessible data source for the BENOPT.

With regard to this use case and the above-mentioned objectives, the BET.db has to fulfil the following requirements: to structure and organise existing bioenergy technology data from different sources, to serve as a machine-readable data source compatible with future extensions, such as NET-technologies. Methodology and results of the associated SOBIO project have been described in recently submitted manuscript [16].

### 2 Basic entities of bioenergy technologies and their relations

The fundamental challenge in storing datasets for bioenergy technologies is primarily to manage complexity, as bioenergy technologies encompass a very wide range of different approaches to handling biomass within a given conversion chain. We attempt to address this issue by identifying the basic building blocks that can be described for most bioenergy technology chains, whether they are linear, branched, complex or simple. We choose to define these building blocks or to use a more general term, entities, along five dimensions and a sixth additional dimension. The first five dimensions of entities represent qualitative information about bioenergy technologies. The parameter dimension is used to describe quantitative properties of the qualitative entities themselves and partly also the quantitative relationships.

The five basic entities include Supply Tasks (ST), Supply Concepts (SC), Conversion Procedures (CP), Process Chains (PC) and Xducts (XD). ST and SC are not bioenergy technologies per se, but have often been used to contextualise bioenergy applications. Energy supply aspects are often a part of energy systems research. CP and PC form the thematic core of the data concept. While the former represents the fundamental transformations of different materials and energy. CP are basic building blocks of PC, but the latter can also be described independently as “black box” PC without specifying the inner CP.

**Fig. 1:**
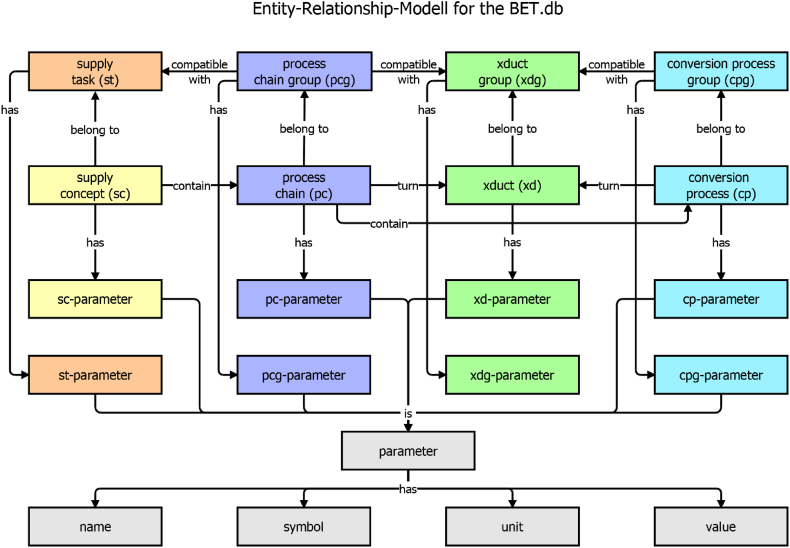
Entity relationship model (ERM), for the main building entities of the Bioenergy Technology Database (BET.db), colours encode domains of supply tasks (orange), supply concepts (yellow), process chains (purple), xducts (green) and conversion processes (cyan).

XD form the connecting entity by covering all types of material and energy exchanges that can flow through or between other entities. These five main building blocks and their interrelationship are represented in an Entity Relationship Model (ERM) (see Fig. 1).

The ERM is constructed in two dimensions. The five main building blocks are arranged in the horizontal dimension, with the exception of the two supply entities, which share one column. The vertical dimension shows the level of aggregation. The top row of entities represents the most general elements described by basic parameters located in the fourth row of Fig. 1. The four groups serve to aggregate the wide variety of individual building blocks. With the exception of the supply concepts, the second row represents individual entities that are linked to more comprehensive parameters located in the third row of Fig. 1. Each parameter in rows 3 and 4 belongs to the parameter entity in the fifth row. Each parameter again is in turn characterised by four sub-characteristics, shown in the sixth row of Fig. 1. This approach allows a very flexible framework to describe a heterogeneous collection of supply, technology, process and xduct entities without static predefinition.

### 3 Database design principles

The design process of the database follows the FAIR guidelines for scientific data management and stewardship [17]. The four letters stand for: Findable, Accessible, Interoperable and Reusable. Findability is ensured by an extensive documentation and precise keyword tagging within this manuscript and for the published dataset at ZENODO [18]. The storage of BET.db on ZENODO also guarantees long-term accessibility. The chosen database format SQLite [19] is very popular, supports the SQL-92 standard defined by the ISO [20,21]. Interoperability as the third pillar of the FAIR principles, is provided by standard SQLite interfaces in many programming languages. SQLite is an open source, stand-alone database format that contains a full SQL database in a single file. There is a range of software available for manually examining and manipulating SQLite databases, most of which is also available under an open source license. An easy-to-use software with a clear graphical user interface is the DB Browser for SQLite [22], which has been used extensively in the developing of BET.db. Reusability is given by the description of the data structure, which is comprehensible and consistent. This requirement was the main challenge for the development of BET.db and is presented in detail in the following sections. Reusability was also a major motivation for the creation of this database. We also decided to publish BET.db under an open licence, and choosing the Creative Commons CC-BY 4.0 international public licence [23].

**Fig. 2:**
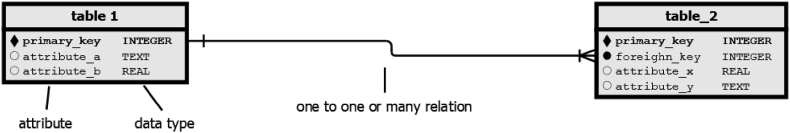
General Approach for connect two data tables in a relational database, where foreign keys pointing to the primary key in another table using crow's foot notation for connector icons. This schema also shows the data type of the individual attributes, which correspond to the columns of the physical data table.

Large and complex data collections can be organised in relational databases, which have specific advantages compared over tree-structured databases [24]. Relational databases use tables to store records of similar content and provide the ability to swap related information to another table (Fig. 2). This approach helps to reduce redundancy and maintain a high level of consistency.

**Fig. 3:**
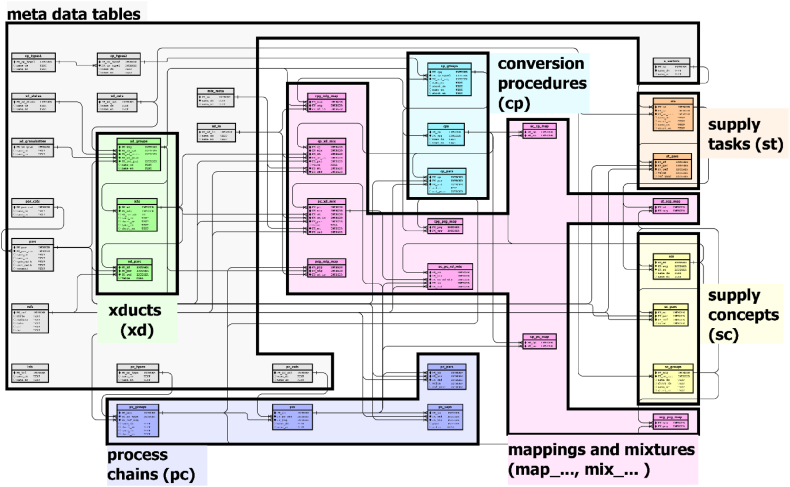
Overview of the database schema with an overlay of the main data table domains referring to the elementary entities given in the ERM (see Fig. 1), a clean (without domains overlay) database schema is given in the supporting information (Fig. S10).

Based on the conceptual ERM (see Fig. 1), the network of connections between more realistic entities was translated into a SQL-compliant database schema. This is a common way of developing such a schema [25].

The ERM contains only the main domains of entities. The derived relational database scheme is much more complex, but still follows the conceptual structure of the ERM.

As shown in Fig. 2 the derived database scheme is more complex than the ERM, but can also be divided into the five main domains of entities (xducts, conversion procedures, process chains, supply tasks, supply concepts) and mapping tables to describe links between tables of different domains. A neat illustration without the overlapping domains can be found in the supplementary information (Fig. S10).

For a deeper understanding of the database contents and inner relationships, the most important tables of the five entity domains are explained in the following subsections.

#### 3.1 Metadata tables

Metadata tables highlighted in grey describe the higher-level context of entities from the domains of xducts, conversion procedures, process chains, supply tasks, supply concepts superordinate entity types or categories. An overview for all metadata tables can be found in Table 1. Metadata tables contain higher-level information for the main domains describing structural relationships and defining hierarchical systematics of these higher levels.

**Table 1:** List of grey-coloured meta-data tables in alphabetical order.

Table name	Content/Function
*cp_types1*	level 1 typing for conversion procedures
*cp_types2*	level 2 typing for conversion procedures
*mix_meta*	meta data for mixes, time relations or reference to product units
*par_cats*	parameter categories
*pars*	parameters
*pc_cats*	categories of process chains
*pc_types*	typing of process chains
*refs*	references for used qualitative data
*s_sectors*	supply sectors as superordinate level for supply tasks
*trls*	list and descriptions of technology readiness levels
*xd_cats*	xduct categories
*xd_granularities*	xduct granularities
*xd_io*	xduct input/output encoding
*xd_*states	states of matter for xducts including energy as a non-material option

For example, *cp_types1*, *cp_types2* and *pc_types* describe CP or rather PC typing for groups of these entities. In contrast to typifying, similar-sounding categories for parameters (*par_cats*), process chains (*pc_cats*) and xducts (*xd_cats*) distinguish fundamental categories of entities. Detailed descriptions of each domain are given in the following sections.

Some of the metadata tables have been used to store global information records. The table *pars* contain a list of parameters, their German and English names and symbols as well as the regular unit of measurement. The unit of the parameters is given in LaTeX notation so that it can be pared directly by programming languages compatible with this syntax, for example the Python library matplotlib [26]. Another widely used table is for storing references (*refs*), which store basic information about cited literature. The consistent use of references should increase the reproducibility and transparency of both qualitative and quantitative values in all data tables.

We have decided not to use the technology readiness levels (TRL) as a static attribute of parameter table. Therefore, the TRL table has no direct relationships to other tables and only stores the TRL definitions. We use the TRL definitions following the basic concept of NASA with TRL from 1 to 9 [27] and the extension for TRL 10 and TRL 11 from the IEA [28].

#### 3.2 Xduct tables

**Fig. 4:**
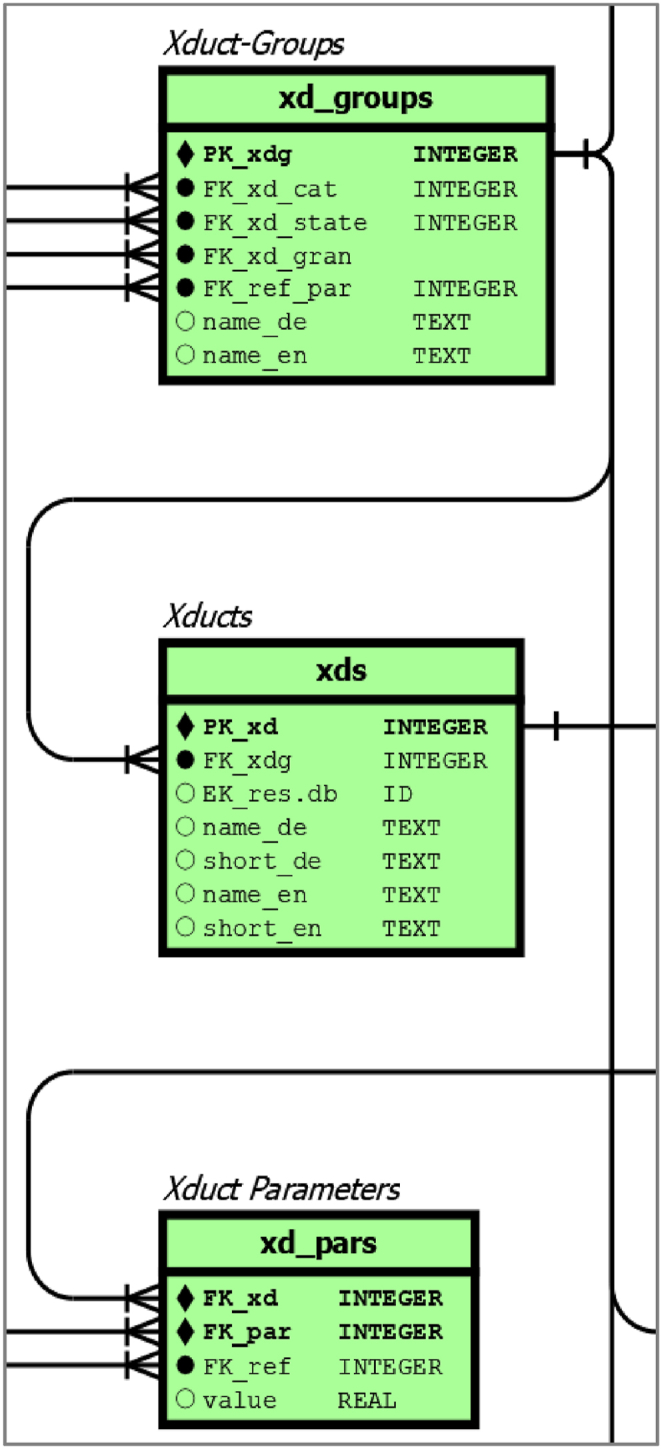
detail of Fig. S10, for green coloured xduct-related tables.

As shown in Fig. 4, which is an enlargement of Fig. S10 **(**see chapter 7**)** green-coloured tables for xducts comprise three main tables: xduct groups (*xd_groups*), xducts (*xds*) and xduct parameters (*xd_pars*). This pattern of an entity group, an entity table and a corresponding parameter table is used in a similar way for most other domains.

The *xd_group* table describes generic elements of xducts without qualitative parameters. This table contains meta information such as the xduct category (*xd_groups.xd_cats*), the regular state of matter (*xd_groups.xd_state*), the granularity (*xd_groups.xd_gran*) and a predefined reference parameter, which is the usual measurement value for the particular xduct-group (*xd_groups.FK_ref_par*). Individual xducts inherit qualitative properties via a foreign key for an xduct group. For individual xducts, the table also contains long and short names in German and English. As a placeholder we also specify a foreign key to the DBFZ resource database, which is currently under construction and is based on the work of Brosowski et al. [29].

In order to achieve a very flexible database design with regard to the parametrisation of the different entities, we create discrete parameter tables, here for xducts. By creating a dedicated qualitative parameter for a particular xduct, a record contains foreign keys for the individual xduct, for the chosen parameter the associated reference and the corresponding value. This approach allows an unlimited number of parameters to be stored for each xduct, while allowing great flexibility in using different parameters for each xduct.

Unlike a fixed table of predefined parameters, there are no data gaps for parameters that were unknown for some xducts provide the opportunity to add more information to existing xducts parameter records.

#### 3.3 Conversion procedures tables

**Fig. 5:**
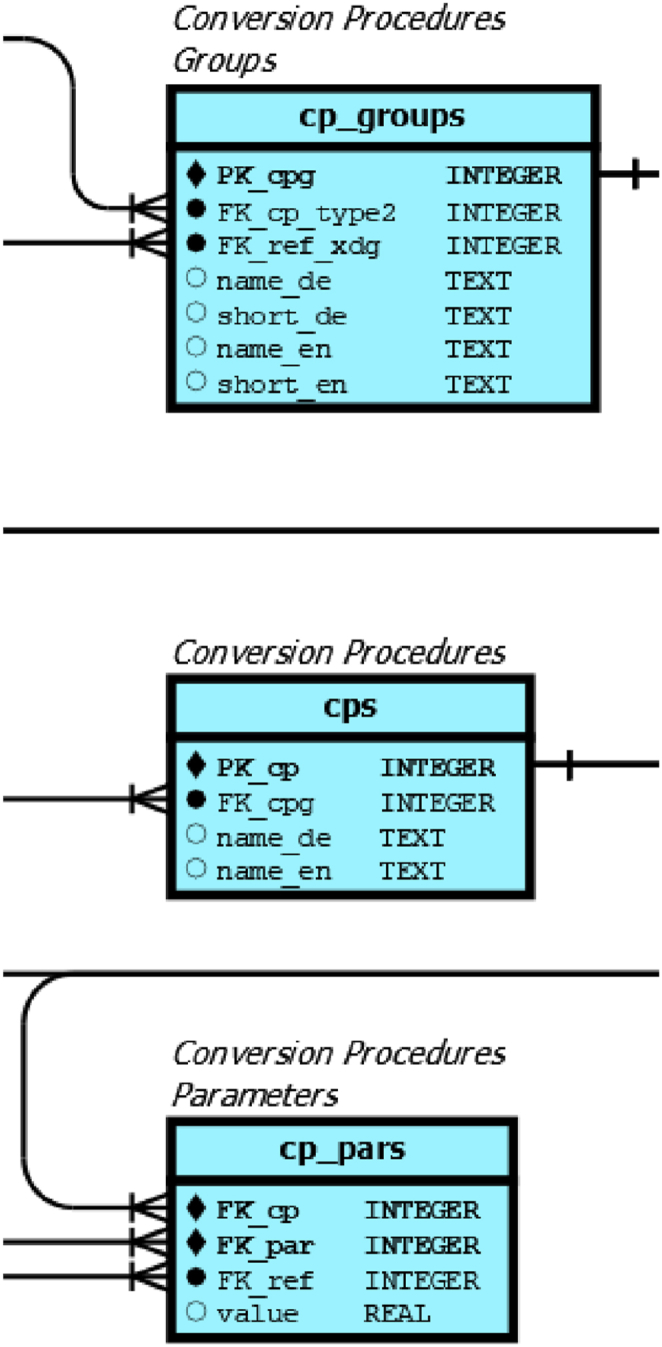
Detail of Fig. S10, for cyan coloured conversion-process tables.

The description of technologies for bioenergy applications is stored in conversion procedures tables (CP) as shown in Fig. 5, an enlargement of Fig. S10 **(**see Chapter 7**)**. They represent the smallest building block of technical entities. The xducts have in *cp_groups* been qualitatively described in terms of metadata tables for CP typing by a corresponding foreign key (FK_cp_type 2). In addition to long and short names, a reference product was assigned to each individual CP group. As with xducts, the records for the individual CP contain only a primary key, which has been defined as an autoincrement integer. In addition, a foreign key for the corresponding CP group and German as well as English names were set.

Similarly to the xduct-table, the parametrisation was outsourced to the *cp_pars* table, where the foreign keys for the individual conversion process, the parameter, the reference used and the value itself are stored. By setting a specific reference year (*cp_pars.ref_year*) it is possible to distinguish parameters for different reference years, for example for changing investment costs or conversion efficiencies. We set *cp_pars.ref_year* to 2022 for all data sets.

For a better human readability and easier query creation, the database already contains a set of VIEWS based on *cps* and the *cp_pars* table, called *V_cps* and *V_cp_pars*. They are not shown in the database schema but are easily accessible by opening the database in a suitable GUI tool, such as DBrowser for SQLite. These VIEWS already include the referenced foreign keys and show the English names of parameter names and units for example for the *V_cp_pars*.

#### 3.4 Process chain tables

**Fig. 6:**
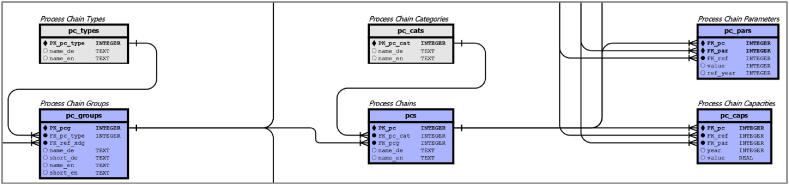
detail of Fig. S10, for dark blue process chain tables.

The detailed view of the PCs in Fig. 6, which is an enlargement of Fig. S10 **(**see Chapter 7**)**, does not only represent individual aggregated conversion procedures, as some process chains contain only one conversion procedure. They can therefore represent blueprints for plant designs, with or without specifications of the included CPs.

If no CPs are specified, the PC can be considered as a black box that can be used as a stand-alone technology. The metadata table (*pc_types*) contains 10 different basic types covering process chains for electricity, heat and fuel production (three sub-types). There are also three types for negative emission technologies and last but one type for undefined technologies. The tables for *pc_groups*, *pcs* and *pc_caps* follow the same paradigm as those for xducts and conversion processes, where in the table *pc_group* only contains a foreign key for the *pc_type*, a foreign key for the reference product and German and English names. Individual process chains were described quantitatively by the *pcs* and *pc_pars* tables. The *pc_pars.ref_year* attribute was set to 2022, but may have different values in the future for assumed parameters. In addition, we add a table to capture current capacities for selected process chains. The *pc_caps* table provides the ability to measure capacities in different units that have been encoded by the foreign key for specific parameters (*pc_caps.FK_par*). As capacities are subject to constant changes, each value is given a time reference to a specific year.

#### 3.5 Tables for supply tasks and supply concepts

**Fig. 7:**
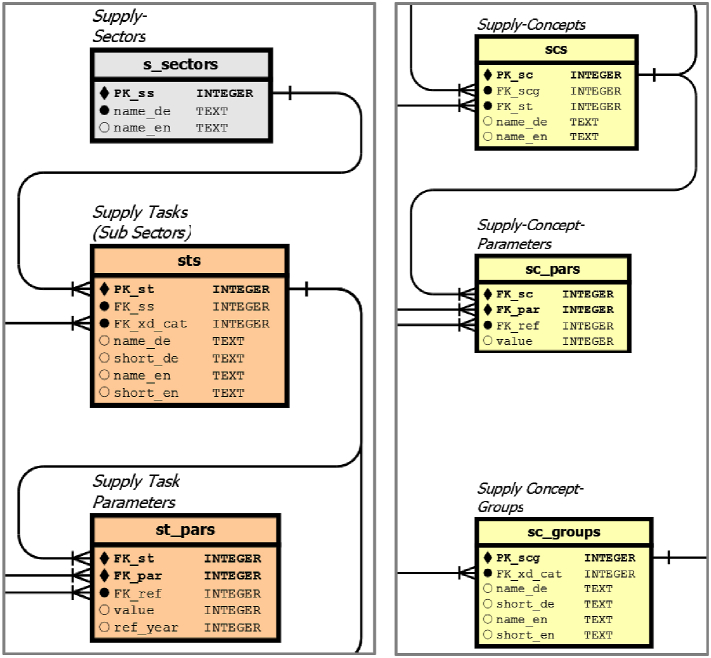
Details of Fig. S10 for orange coloured supply task (left) and yellow coloured supply concept (right) tables.

Supply Tasks (ST) and Supply Concepts (SC) have been used to describe typical applications of bioenergy technologies, although they do not strictly belong to technology entities such as xducts, conversion processes and process chains (Fig. 7, represent two enlargements Fig. S10, see chapter 7).

ST describe sectoral energy demands usually defined by a dataset of ST parameters. In particular, we collect ST for heat sinks and using the total annual demand, annual full load hours and peak demand. As with the *cp_pars* and *pc_pars* table, *st_pars.ref_year* has been set to 2022 but may have different values for historical or future supply scenarios.

They are also organised by a qualitative grouping table (*s_sectors*) and a set of general properties for the supply sector corresponding to the xduct category (*s_sectors.xd_cat*) and the long and short names.

The SC tables (*scs*, *sc_pars*, *sc_groups*) contain information on general properties. For example, heat supply concepts have an auxiliary power demand, installation and maintenance costs.

At the current stage of development, ST and SC focus on heat supply, as the power and fuel markets are often structured in a larger scale, while heat is more locally due to its poor suitability for long-distance transport. Multivalent concepts also play an important role in the supply of heat from renewable sources, which was one of the sectors analysed in the study mentioned above [15]. Multivalent concepts make supply concepts more complex than traditional monovalent solutions and therefore require an appropriate disaggregation for this database.

#### 3.6 Mapping tables

Mapping tables serve as a link for relationships between entities of different domains, thus mapping the structural context within the bioenergy nexus. According to Table 2, six mapping tables provide a qualitative context between different tables of the main technology domains. As mapping tables are a core element for describing structural relationships, we have explained them individually in the following paragraphs in alphabetical order.

**Table 2:** List of pink-coloured (see Fig. 3 and 10) mapping tables.

Table name	Connected table #1	Connected table #2
*cp_pc_map*	*cps* (conversion processes)	*pcs* (process chains)
*cpg_pcg_map*	*cp_groups* (process chain groups)	*pc_groups* (conversion procedure groups)
*cpg_xdg_map*	*cp_groups* (conversion process groups)	*xd_groups*
*pcg_xdg_map*	*pc_groups* (process chain groups)	*xd_groups*
*sc_pc_map*	*scs* (supply concepts)	*pcs* (process chains)
*scg_pcg_map*	*sc_groups* (supply concept groups)	*pc_groups* (process chain groups)
*st_scg_map*	*sts* (supply tasks)	*sc_groups* (supply concept groups)

The table *cp_pc_map* provides a link between CPs and PCs. This function makes it possible to describe PCs in a finer granularity and to indicate which individual procedures form a relevant conversation chain. We have not yet made use of this option, but we will implement this function to enable the possible deconstruction of PCs. The *cpg_pcg_map* table has a similar function to the table *cp_pc_map*, but shows the relationship between CPs and PCs at the group level to define a general relationship between the basic group entities.

The table *cpg_xdg_map* describes the interoperability between CP groups and xduct groups, with foreign keys for CP groups (*cpg_xdg_map.FK_cp_group),* XD groups (*cpg_xdg_map.FK_xd_groups*) and for indicating whether the xduct group acts an input or an output. The table *pcg_xdg_map* follows the same paradigm but refers to PCgroups instead of CP groups.

The table *sc_pc_map* defines the corresponding process chains used in a given SC, regardless of the exact contribution of each PC to meeting the demand of the SC. This aspect is covered by the table *sc_pc_xd_mix* described in the next section. The table *scg_pcg_map* play a similar role, but operates at the group level for SC and PC, thus defining a more general relationship between these entities.

The table *st_scg_map* links the ST to the SC groups, so that multiple SC groups can be defined for a single ST. Detailed information on how to parameterised SC is encoded in the SC table (*scs*).

#### 3.7 Mix tables

Mix tables are the links to specify quantitative relationships between different entities and they describe mass and energy flows both for CPs and for PCs (Table 3), .

**Table 3:** List of pink-coloured (seFig. 3 and 10) mix tables and the connected primary or secondary data tables respectively.

Table name	Connected table #1	Connected table #2
cp_xd_mix	cps (conversion processes)	xds (xducts)
pc_xd_mix	pcs (process chains)	xds (xducts)
sc_pc_xd_mix	scs (supply concept)	pc_xd_mix (process chain-xduct-mix)

**Fig. 8:**
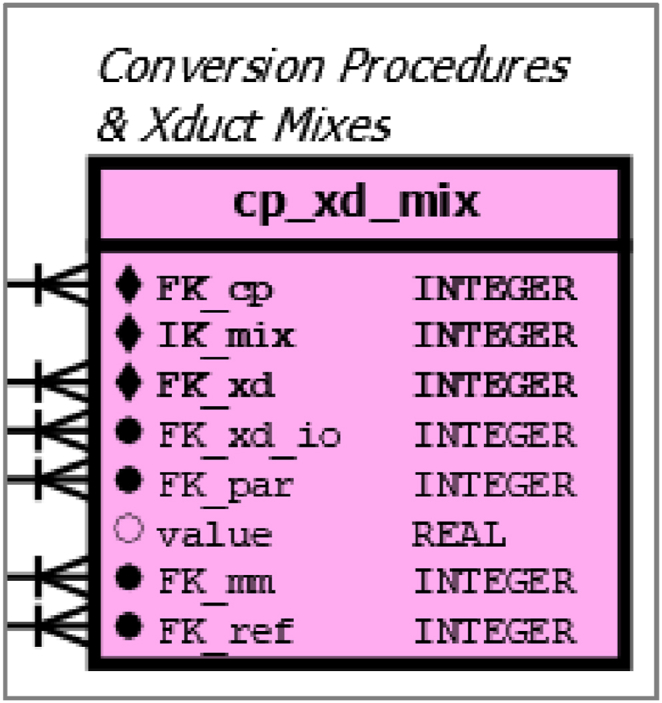
detail of Fig. S10, Mix-table for conversion procedures and xducts.

At CP level, the table *cp_xd_mix* stores the information for defined mixtures at different modes of operation (Fig. 8). The table has a composite primary key, indicated by the diamonds in Fig. 8. Such a single record is uniquely identified by three elements: 1.) The foreign key to the CP (cp_xd_mix.*FK_cp*). 2.) The index key (cp_xd_mix.*IK_mix*) for the respective mix of a given combination of a CP and a XD. 3.) The foreign key for the XD (cp_xd_mix.*FK_xd*). The individual attributes of a data set consist of foreign keys for xduct input-output parameters (cp_xd_mix.*FK_xd_io*), the meta information for the selected parameter (cp_xd_mix.*FK_par*), a temporal reference attribute (cp_xd_mix.*FK_mm*), the reference to the primary source (cp_xd_mix.*FK_ref*) and finally the numerical value (cp_xd_mix.value). As mixtures can refer to different time periods, cp_xd_mix.*FK_mm* encodes the time context of the energy or mass turnover. The table *mix_meta* contains the following contexts: 'per full load hour’, ‘per annual use hour’, ‘per year’ or as a non-temporal reference ‘per product unit’. This approach should make it possible to store mixtures of different temporal references. There is no common usage for the different bioenergy sectors in the literature and the database should be able to cover different types of technology specification.

**Fig. 9:**
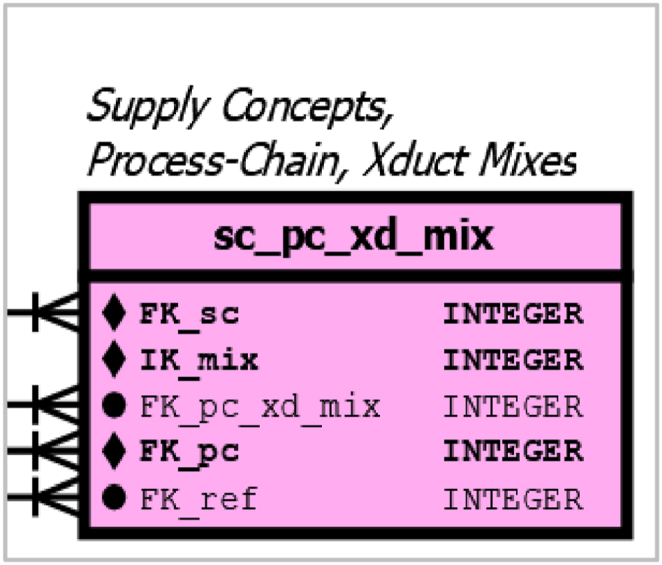
detail of Fig. S10, mix table for supply-concepts and the mix table for process chains as well as xducts.

The table *pc_xd_mix* is very similar to the table *cp_xd_mix*, except that the first attribute uses the foreign key for PC (*pc_xd_mix.FK_pc*) instead of CP (*cp_xd_mix*.FK_cp), see Fig. 9, which is an enlargement of Fig. S10.

The separate table allows the definition of mixtures for the higher aggregated entities (PC), for which there is no data or no need for finer granularity of the subdivided PC.

The table *sc_pc_xd_mix* is a higher-level mix table in which foreign keys for SC (*sc_pc_xd_mix.FK_sc*) are linked to already defined records of PC-XD mixtures (*sc_pc_xd_mix.FK_pc_xd_mix*). As can be seen in Fig. 9, this mix table also uses a composite primary key, which has not been ordered in the graphical schema for a cleaner arrangement of the link lines. The use of two foreign keys (*sc_pc_xd_mix.FK_pc_xd_mix* and *sc_pc_xd_mix.FK_pc)* link to the corresponding rows in the PC-XD mix table. However, since the table *pc_xd_mix* also use a composite primary key consisting of *pc_xd_mix.FK_cp*, *pc_xd_mix.IK_mix* and *pc_xd_mix.FK_xd*, all are needed to identify the corresponding rows of *sc_pc_xd_mix*.

#### 3.8 Predefined VIEWS

For better (human) readability and easier query creation, the database contains a set of fourteen VIEWs (Table 4). VIEWs are table-like elements that represent the results of a predefined query and contain data collected and combined from different tables. The VIEWs are not shown in Fig. S10, a brief description of them were given in Table 4. Each VIEW consists of both English and German meta data attributes and selected quantitative attributes.

**Table 4:** List of VIEWs, not portrayed in the graphical database schema (Fig. S10).

View	Content
*V_cp_pars*	view for parameters of conversion procedures (CP)
*V_cp_types*	view for CP types including type 1 and type 2 attributes
*V_cpg_xdg_map*	*view for the mapping of conversion procedure-groups and xduct-groups*
*V_pars*	*view for the parameters, which are already defined*
*V_pc_pars*	*view for parameters of processes chains*
*V_pc_xd_mix*	*view for mixtures of process chains and xducts with explicit values*
*V_pcg_xdg_map*	view for mapping of process chain groups and xduct groups
*V_pcs*	view for individual process chains
*V_sc_pars*	view for parameters of supply concepts
*V_sc_pc_xd_mix*	view for mixtures of supply concepts, process chains and xducts
*V_st_pars*	view for supply task parameters
*V_st_scg_pcg_map*	view for mapping of supply tasks, supply concepts and process chains
*V_xd_pars*	view for xduct parameters
*V_xds*	view for xducts

#### 3.9 Key figures

For the current stage of development BET.db consists of 41 tables with a total number of 6,908 records, of which 3,413 are quantitative records for the different entities. The main entities were represented by 352 conversion procedures, 259 process chains, 18 supply tasks, 134 supply concepts and 141 xducts.

### 4 ETL methods for data processing

In computing, extract, transform, load (ETL) is a three-stage process in which data are extracted, transformed (cleaned, sanitised, scrubbed) and loaded into an output database [30]. Here we have collected raw datasets mainly from the three supplementary materials of the studies presented in the introduction. In addition, we performed web searches to enrich the dataset with information on technologies and products, needed for the SOBIO project and not yet covered by the existing studies.

We decided to automate the ETL-process using Python scripts, as even the raw data sources used contain several inconsistencies, leading to many exceptions and corrections.

#### 4.1 Extraction

As all the supplementary materials were available in MS EXCEL format we used the openpyxl library to extract information from the raw data files. The use of openpyxl allowed a very precise tabular extraction for the heterogeneous raw data files. We program two different functions with respect to the different data structures in the supplementary material from Lenz et al. [11] and Meisel et al. [13].

The supplementary material from Lenz et al. is an MS Excel file (Tech-Cost-Data-01-2019opendata.xlsx) with 19 worksheets, each representing one of the supply concepts examined in their study. Each worksheet contains a header section with a description and some key figures of the supply task. It also contains descriptions of the supply concepts associated with the supply task. The technical key figures were arranged in a data table below the header section, with each supply concept accounting for one column in the table. Since each supply task has a different selection of supply concepts, we needed to program an extraction routine, that recognises the number of supply concepts and reads the exact number of columns from the data table. During the data extraction we derive metadata for the supply concepts as well as the technical end economic key values.

However, the supplementary material from Meisel et al. contains only a single data table within the MS Excel file (BKS_Quote_et_al_SOBIO.xlsx). Extracting data from this single spreadsheet, was much easier as the attributes for technologies examined were arranged simply in rows and 51 columns.

#### 4.2 Transformation

The extracted raw datasets were subjected several transformations, including verification tests and consolidation for redundant data. We only describe the main procedures because the datasets contain an unexpectedly high number of inconsistencies, requiring several exceptions and error handling within the transformation functions.

Simple transformations were used to convert energy units, as there was a mixture of joule, watthour or sometimes just biomass dry matter. The first two units can easily converted, but for conversion of biomass into energy we also need to collect data for the assumed energy content of these energy carriers.

Another major problem was that, particularly in Lenz et al. file, the naming of technologies, energy carriers and supply concepts was not consistent. For example, each technology was represented by an average of 4.2 aliases, with a maximum of 8 aliases for a single technology ("pellet boilers"). The description of the key values also needs to be streamlined, for which 11 individual attributes were mapped, with an average of 3.8 aliases per attribute and a maximum of 12 aliases for a single attribute ("thermal capacity").

We also had to define a number of exceptions to the programmed transfer functions, as the dataset also contained some errors that couldn't be corrected using standard procedures.

The Meisel et al. dataset doesn't require as much translation because the single data table was already quite consistent. We only need to translate some of the technical values into the target unit system of BET.db. For example, many of the economic values were given in relative proportions to the initial investment costs of the technologies. Therefore, we calculate the absolute values of maintenance and operating costs based on the investment. Although the dataset was largely consistent, one minor issue was that investment costs were given in € per kW of installed capacity, but the capacity was given in terms of the annual output of the main product.

#### 4.3 Loading

Loading extracted and transformed data has been added to the Python data processing routines using SQL statements like INSERT or UPDATE, which can be utilised via the standard python library for SQLite (sqlite3). The Python functions work incrementally, loading individual data points into the database. Since the data structure of the input files differs from the data structure in the database, the extracted information was reassigned to the new data structure in BET.db.

### 5 Discussion

The approach outlined for collecting and structuring data on bioenergy technologies was the first attempt to combine a number of existing data sources into a comprehensive data collection. Although we have collected a large amount of data, BET.db contains only selected bioenergy technologies that are of particular interest for the connected SOBIO project. BET.db can therefore be seen as a first prototype that can serve as a starting point for the further development of a growing collection of bioenergy technologies. There are already plans to transfer the concept of BET.db and the existing datasets to a web application, in order to provide easier access to bioenergy technology data and to have the possibility to incorporate new elements. Thus, BET.db will be further developed in the near future to create a server-based SQL-database, different from the SQLite format, coupled with a feature-rich front-end. We want to lower the barriers to using the database for a wider range of users who may struggle with SQL syntax or the version control challenges of using a single file format.

Regarding the structural design of the database, a derivation of the table *sc_pc_xd_mix* for SC and single CP (new table: *sc_cp_xd_mix*) would be a possible addition if energy and mass flows are to be described at the disaggregated level of CPs. So far, we have decided not to include this additional table in order to limit the complexity of the current database structure. As with table sc_*pc_xd_mix*, the optional extension of *sc_cp_cd_mix* would allow to store different compositions of SC energy flows specifying detailed conversation procedures.

Proof of data integrity and consistency was provided by running SQLite on-board tools. To test the integrity of the database, the SQL command: PRAGMA integrity_check was executed and the consistency of the foreign keys was checked by executing the SQL command: PRAGMA foreign_key_check.

Performing the integrity check was sccessful but foreign key error gives an error message for a foreign key mismatch of *sc_pc_xd_mix* referencing *pc_xd_mix*. The error is triggered by using a single foreign key in *sc_pc_xd_mix.FK_sc_xd_mix* pointing to *pc_xd_mix.IK_mix* to reference already defined mixtures associated with shared process chains in both tables.

Since *pc_xd_mix* uses a composite primary key consisting of four attributes (*pc_xd_mix.FK_pc, pc_xd_mix.IK_mix, pc_xd_mix.FK_xdm, pc_xd_mix.FK_xd_io),* a correct foreign key should also have four elements to uniquely reference records in the referenced table. We decide to leave this small logical inconsistency but maintain the nomenclature for attributes, here using of the substring " FK_" to indicate that this attribute points to another table.

In the predefined view *V_sc_pc_xd_mix,* the relevant tables have been used to create a common tabular data fusion, which works correctly, even though it does not meet the logical requirements for foreign keys in a relational database.

We are also working intensively with the database as a data source for modelling optimal cross-sectoral biomass allocation using the BENOPT model [31], based in ’MATLAB's SQL interface to directly import the required datasets into the BENOPT model. In addition to a logical check of the structural integrity, we can also demonstrate the practical integration of BET.db for a typical use case. From the modellers point of view this approach greatly simplified the data acquisition process and ensured that the generated results are fully referenced to the primary data origin through the integrated metadata information.

The total number of 6,908 datasets is distributed over 36 tables. The tables *pc_caps*, *cpg_pcg_map* and *cp_pc_map* are still empty, as the use case within the SOBIO project focuses on aggregated PCs and not on individual CPs. However, these tables are prepared to handle disaggregated PCs or individual CPs in the near future.

Although the current stage of development stage of the database does not explicitly include Negative Emission Technologies (NET), the data structure described has been carefully designed to be NET-ready. NET will be an important complementary aspect of many PCs covering bioenergy [32]. We have discussed extensively that NET do not have to be technical solutions as commonly associated with 'technology' in the literal sense. NET also include land use systems that enable carbon sequestration, which should also fit into BET.db. The structure of the database therefore also allows the description of STs for negative emissions in t CO_2_. As with classical STs, the database can be used to define SCs, by using one or many PCs, which can also contain NET options. The NET options themselves can be considered as PCs as CPs. For example, a frequently discussed NET option is the rewetting of degraded peatlands. This option can be categorised as a CP with one main product, namely the assumed sequestration rate for a given area of peatland over a given period of time. Thanks to discussions with colleagues participating in the BioNET project [33], we have already created some meta information on NET options with specific groups for XDs, CPs and PCs, as well as some basic parameters to allow an immediate implementation of explicit NET.

### 6 Conclusion and Outlook

The presented approach to construct an SQL database schema for the very heterogeneous field of bioenergy and bioeconomy technologies follows the concept of atomising the different entities. The resulting data structure achieves a high degree of flexibility for adding new technologies, whose specific attributes and relations to existing entities are still unknown. The proof of concept has been successfully realised in modelling different bioenergy scenarios in net zero energy system scenarios for Germany. The scenarios cover a wide range of technologies for fulfil different supply tasks in the power, heat and transport sector. The subsequent implementation of the NET-readiness also shows that the basic design allows a flexible extension and further propagation of the database for future developments.

A major disadvantage of the atomisation approach is that the database scheme becomes very complex. Not only in terms of the absolute number of data tables, but also in terms of the multiple links between these tables. Especially in the case of mapping and mixture tables, where the information link is usually established by combining different foreign keys, many references are merged. This is not necessarily an obstacle to using the database in a production environment. Data exchange is usually organised by scripted routines based on SQL syntax, but it can be difficult to think one way into the data structure when imagining where to find or store data.

However, with the help of this manuscript, which provide clear guidelines for understanding and using the database, we expect a huge potential to implement the database for future energy modelling to improve the technological granularity of the bioenergy domain.

Furthermore, the BET.db allows the scientific community to access a large dataset of raw materials, conversion technologies and energy carriers and to save the effort of searching for key figures for bioenergy when preparing energy system studies. In particular, especially studies that, which consider bioenergy technologies can benefit from the prepared data source, which can also be extended for individual requirements. Compared to the existing raw data sources, especially those formatted in MS Excel the BET.db offers a solution with a simple machine-readable interface, that allows a more granular consideration of bioenergy. It is planned to realise a web application based on BET.de in 2024. This web application will offer an option for uploading new data sets and an Application Programming Interface (API).

In many recent studies the inherent complexity of bioenergy often leads to a simplified approach, but at the cost of losing a realistic model of the bioenergy nexus. By providing a one-stop-shop solution for techno-economic information about on the bioenergy nexus, this blind spot can be avoided for further investigations.

### CRediT authorship contribution statement

**Martin Dotzauer:** Writing – original draft, Visualization, Validation, Software, Methodology, Investigation, Formal analysis, Data curation, Conceptualization. **Kai Sven Radtke:** Writing – review & editing, Validation, Methodology. **Matthias Jordan:** Writing – review & editing, Validation, Investigation. **Daniela Thrän:** Writing – review & editing, Supervision, Funding acquisition.

## Declaration of competing interest

The authors declare that they have no known competing financial interests or personal relationships that could have appeared to influence the work reported in this paper.

## Figures and Tables

**figs1:**